# Anterior uveitis associated with laser epilation of eyebrows

**DOI:** 10.1186/1869-5760-3-45

**Published:** 2013-04-15

**Authors:** Fatime Nilüfer Yalçındağ, Aslıhan Uzun

**Affiliations:** 1 Department of Ophthalmology, Faculty of Medicine, Ankara University, Mamak Cad. Dikimevi, Ankara, 06100, Turkey

**Keywords:** Alexandrite laser, Laser epilation, Anterior uveitis

## Abstract

**Background:**

The objective of the study is to report a case of unilateral anterior uveitis after laser hair removal of the eyebrows with an alexandrite laser.

**Findings:**

A 36-year-old female presented with painful red eye and photophobia in her left eye 2 days after receiving alexandrite (755 nm) laser epilation of both eyebrows. Visual acuity was 20/20 in both eyes. Right eye examination was normal. Left eye examination showed conjunctival injection, 2+ cells in the anterior chamber, and local posterior synechiae. Intraocular pressure and fundus examination were normal in both eyes. Topical steroids and cycloplegic drops were prescribed. Three days after the initiation of topical treatment, there was a reduction in anterior chamber cells to 1+, but posterior synechiae was enhanced. One week after, there were 0.5+ cells in the anterior chamber and no further enlargement of posterior synechiae. At the 2-month follow-up, uncorrected visual acuity remained 20/20 in both eyes. Slit-lamp biomicroscopy of the right eye was normal. Intraocular pressure and fundus examination were still normal in both eyes. Although anterior chamber of the left eye was clear, posterior synechiae persisted. We are still following the patient.

**Conclusion:**

Laser hair removal of the eyebrows can lead to ocular damage and should be avoided.

## Findings

### Introduction

Laser-assisted hair removal is currently the most popular and efficient method for long-term removal of unwanted body and facial hair. Various laser systems including red spectrum ruby laser (694 nm), near-infrared spectrum alexandrite laser (755 nm), diode laser (800 nm), neodymium:yttrium-aluminum-garnet (Nd:YAG) laser (1,064 nm), and intense pulse light (590 to 1,200 nm) are available for this use. Most of the time, more than one session is needed. Ocular protection with protective glasses during laser hair removal is recommended. Most complications of laser epilation such as crusting and vesiculation of the treatment site, hypopigmentation, or hyperpigmentation are not generally serious [1,2]. Periocular laser epilation is rarely associated with complications. Although various case reports of ocular complications with laser epilation of eyebrows have been described in the literature, most of these reports revealed ocular complications with diode laser [3-6]. In this paper, we describe a case of anterior uveitis caused by alexandrite (755 nm) laser-assisted removal of eyebrows.

### Case report

A 36-year-old healthy female with no history of ocular problems had undergone alexandrite (755 nm) laser-assisted hair removal of both eyebrows. She stated that she had covered her eyes with one finger during the procedure but had not used protective glasses because of the difficulties caused by their size. Although there were no problems during laser epilation of the right eyebrow, the patient indicated that she felt a temporary pain in her left eye during the treatment. Two days after receiving laser epilation, she presented to our clinic with the complaints of severe pain, redness, and photophobia in her left eye. On ophthalmologic evaluation, uncorrected visual acuity was 20/20 in both eyes. Slit-lamp biomicroscopy of the right eye was unremarkable. There were moderate conjunctival injection, 2+ cells in the anterior chamber, and local posterior synechiae in her left eye. Intraocular pressure and fundus examination were normal in both eyes. Topical dexamethasone (eight times per day) and cyclopentolate hydrochloride eyedrops (three times per day) were prescribed. Three days after the initiation of topical treatment, there was a reduction in anterior chamber cells to 1+, but posterior synechiae was enhanced. A subconjunctival injection of adrenaline and dexamethasone was administered. Three days after the injection, the anterior chamber cells reduced to 0.5+, and there was no further enlargement of the posterior synechiae. Topical dexamethasone eyedrops were tapered to five times per day, but cyclopentolate hydrochloride eyedrops were continued. One week later, the anterior chamber was clear, but the posterior synechiae persisted in her left eye. Topical medications were gradually tapered and then stopped. Additionally, she was told not to receive laser epilation of eyebrows. At the 2-month follow-up, uncorrected visual acuity remained 20/20 in both eyes. Slit-lamp biomicroscopy, intraocular pressure, and fundus examination of the right eye were still unremarkable. Slit-lamp biomicroscopy of the left eye revealed a clear anterior chamber and persistent posterior synechiae. Intraocular pressure and fundus examination of the left eye remained normal. Follow-up examinations of the patient are still continuing in our department.

### Discussion

Laser epilation, based on selective photothermolysis, is achieved through follicular unit destruction. Melanin in the hair follicles at the dermis level is the target of laser-assisted systems. Although treatment outcomes are not affected by the laser system preferred, laser parameters are important when choosing the appropriate system for the patient. The shorter the wavelength, the more the laser energy may cause epidermal damage and side effects [2]. Alexandrite and diode lasers do not affect the more superficial epidermal tissue and were designed to prevent potential side effects by penetrating deeper layers of the skin. The penetration depth of these laser systems is approximately 3 to 4 mm.

The probe must be firmly placed on the skin during the procedure, but the structure of orbital rim makes this difficult when used on the hair of eyebrows. Additionally, in most cases, the patients are told to remove the protective glasses because of their large size. Furthermore, the normal Bell’s phenomenon causes the pigmented iris to be damaged by the peripheral laser beams. The thin skin of the eyelids fails to prevent the penetration of the laser beams to the eye; thus, a variety of clinical presentations can occur with laser epilation of eyebrows. Lin et al. and Carrim et al. reported patients with pigment dispersion and temporary increased intraocular pressure associated with alexandrite laser epilation of eyebrows [7,8]. Elkin et al. described a 41-year-old male who presented with bilateral iritis and iris atrophy without posterior synechiae 2 days after receiving alexandrite laser epilation of eyebrows [9]. Our case is different from their case because of the presence of posterior synechiae without iris atrophy.

The laser beams do not appear to penetrate deeply enough to reach the posterior segment of the eye. Accordingly, previous case reports revealed normal fundus appearance in cases with anterior segment side effects associated with laser hair removal of eyebrows [4,5,7,9]. Nevertheless, Sheikh et al. described a 38-year-old woman who presented with unilateral acute anterior uveitis and a diffuse peripheral visual field defect after receiving diode laser hair removal of eyebrows with protective metal goggles. Although fundus examination was normal, the electroretinography of this patient was abnormal, suggesting that there was a dysfunction of the photoreceptor cells in the peripheral retina [6].

Despite the use of less penetrating alexandrite laser, ocular damage can occur with laser-assisted hair removal of eyebrows. Consequently, the procedure should not be suggested as a simple method, and individuals contemplating laser epilation of eyebrows should be warned against the potential risks. If the patient is decisive in having the procedure, well-educated and experienced physicians should be chosen, and protective glasses should be worn. In case of ocular pain during laser treatment, the procedure should be stopped and the patient should be referred to an ophthalmologist.

## Consent

The patient gave consent to publish individual data.

## Competing interests

The authors declare that they have no competing interests.

## Authors’ contributions

FNY conceived of the study, participated in its design and coordination, and revised the manuscript critically for important intellectual content. AU participated in the design and coordination of study and drafted the manuscript. Both authors read and approved the final manuscript.
